# Protective Effect of Aqueous Centella asiatica Extract on Ethanol-Induced Gastric Mucosal Injury: Short title: Centella asiatica Protects Against Ethanol-Induced Gastric Injury

**DOI:** 10.31661/gmj.v15i.4247

**Published:** 2026-08-15

**Authors:** Nzar Ibrahim Yousif, Zhala B Taha, Mohammed M. Hussein M. Raouf, Bakhtawar Ziad Omer, Zahraakhan Maaroof Taher, Abdullah Jawdat Zebari, Zeenath S Sheikh, Hero Ismael Mohammed, Shahrokh Mojarradgandoukmolla, Tanya Salam Salih, Usha Rani Kandula, Sherzad Hassan Radha, Abdulrahman Ismael

**Affiliations:** ^1^ Department of Community Health Nursing, Cihan University-Erbil, Erbil, Kurdistan Region, Iraq; ^2^ Department of Forestry, College of Agricultural Engineering Science, Salahaddin University-Erbil, Erbil, Kurdistan Region, Iraq; ^3^ Department of Biology, Faculty of Science and Health, Koya University, Koya 44023, Kurdistan Region, Iraq; ^4^ Department of Biomedical Sciences, Cihan University-Erbil, Erbil, Kurdistan Region, Iraq; ^5^ Department of Medical Biochemical Analysis, Cihan University-Erbil, Erbil, Kurdistan Region, Iraq; ^6^ Department of Anesthesia Technology, Cihan University-Erbil, Erbil, Kurdistan Region, Iraq; ^7^ Department of Biology, College of Education, Salahaddin University-Erbil, Erbil, Kurdistan Region, Iraq; ^8^ Department of Physiotherapy, Cihan University-Erbil, Erbil, Kurdistan Region, Iraq

**Keywords:** Centella asiatica, Gastric Ulcer, Ethanol-Induced Gastric Injury, Nrf2/HO-1 Pathway, Gastroprotection, Oxidative Stress

## Abstract

**Background:**

Gastric ulcer disease is associated with oxidative stress, inflammation, and disruption of gastric mucosal defense mechanisms. Ethanol-induced gastric injury is a widely used experimental model for evaluating gastroprotective agents. Centella asiatica is a medicinal plant rich in bioactive phytochemicals, including flavonoids, phenolic compounds, saponins, and triterpenoids, which possess antioxidant and anti-inflammatory activities.This study evaluated the gastroprotective effect of aqueous C.asiatica extract against ethanol-induced gastric mucosal injury in rats.

**Materials and Methods:**

Adult male Sprague-Dawley rats were divided into five groups (n=6): ulcer control, omeprazole-treated group (20 mg/kg), and three groups pretreated orally with aqueous C.asiatica extract at doses of 150, 300, and 600 mg/kg. Preliminary qualitative phytochemical screening of the extract was performed using standard methods. Gastric ulceration was induced using absolute ethanol (5 mL/kg). Gastric pH, mucus weight, ulcer area, and ulcer inhibition percentage were evaluated. Histopathological examination and quantitative real-time PCR analysis of Nrf2 and HO-1 expression were also performed.

**Results:**

Phytochemical screening confirmed the presence of flavonoids, phenolic compounds, saponins, and triterpenoids in the aqueous C.asiatica extract. Pretreatment with the extract significantly reduced ethanol-induced gastric injury in a dose-dependent manner (P<0.05). Treated rats showed increased gastric pH and mucus weight with reduced ulcer area compared with the ulcer control group. The 600 mg/kg dose produced the highest ulcer inhibition (98.88%) and preserved gastric mucosal architecture with minimal epithelial disruption, edema, and inflammatory-cell infiltration. In addition, the extract significantly upregulated Nrf2 and HO-1 expression levels.

**Conclusion:**

Aqueous C.asiatica extract demonstrated significant gastroprotective activity against ethanol-induced gastric mucosal injury in rats, possibly through enhancement of mucosal defense mechanisms and activation of the Nrf2/HO-1 antioxidant pathway. The observed protective effects may be attributed to the phytochemical constituents present in the extract, supporting the potential therapeutic value of C.asiatica in gastric ulcer prevention.

## Introduction

Peptic ulcer disease (PUD) remains a significant global health problem despite advances in acid-suppression therapy and eradication of Helicobacter pylori [1]. In addition to gastric acid and pepsin activity, current evidence highlights oxidative stress, inflammatory injury, microvascular dysfunction, and impairment of mucosal defense mechanisms as major contributors to ulcer development and delayed healing [2, 3]. Ethanol-induced gastric injury is widely used as an experimental model because it reproduces important pathological features, including epithelial exfoliation, submucosal edema, hemorrhagic necrosis, and inflammatory-cell infiltration, while simultaneously suppressing endogenous mucosal defense systems such as mucus-bicarbonate secretion and prostaglandin-mediated cytoprotection [1, 2, 3].

The balance between gastric mucosal injury and protection is strongly influenced by redox-sensitive signalling pathways [4]. Nuclear factor erythroid 2-related factor 2 (Nrf2) is an important transcription factor that regulates antioxidant defence through activation of antioxidant-response element (ARE)-dependent genes, including hem oxygenase-1 (HO-1) [4, 5]. Activation of the Nrf2/HO-1 pathway contributes to reduction of oxidative stress, preservation of mitochondrial function, and maintenance of epithelial-cell survival. In addition, cyclooxygenase-2 (COX-2)-derived prostaglandins play an essential role in maintaining gastric mucosal integrity by supporting mucus and bicarbonate secretion, improving mucosal blood flow, and facilitating tissue restitution following injury [4, 5, 6]. Therefore, therapeutic strategies that enhance antioxidant defense and mucosal protection may reduce gastric injury and improve healing [6] .

Centella asiatica (L.) Urb. (Apiaceae), commonly known as gotu kola, is a medicinal herb traditionally used in Asian and African medicine for wound healing, inflammatory disorders, and neurological conditions [7]. The plant contains several bioactive constituents, including triterpenoid saponins (asiaticoside and madecassoside), asiatic acid, madecassic acid, flavonoids, and phenolic compounds [7, 8]. These phytochemicals have demonstrated antioxidant, anti-inflammatory, angiogenic, and tissue-repair properties in previous experimental studies [7, 8, 9]. Earlier investigations have also suggested that C. asiatica may promote epithelial regeneration, reduce oxidative injury, and attenuate inflammatory-cell infiltration, indicating its potential role in gastric mucosal protection [7].

Proton-pump inhibitors (PPIs), including omeprazole, remain standard therapeutic agents for gastric ulcer management because they effectively suppress gastric acid secretion and elevate intragastric pH [10]. However, acid suppression alone may not fully address oxidative and inflammatory mechanisms involved in ethanol-induced gastric injury. Therefore, plant-derived agents capable of enhancing mucosal defence while modulating oxidative stress and inflammatory pathways may provide additional gastroprotective benefits [10, 11, 12, 13]. The gastroprotective activity of an aqueous extract of C.asiatica was investigated in ethanol-induced gastric injury in rats [14, 15, 16, 17, 18]. Gastric pH, mucus production, ulcer area, histopathology, and Nrf2/HO-1 expression were evaluated. This study examined whether the extract attenuates gastric damage via reinforcement of mucosal defences and activation of antioxidant pathways.

## Materials and Methods

### Preparation of Centella asiatica Extract and Dose Formulation

Fresh aqueous extract of C.asiatica was prepared daily throughout the experimental period. Three doses of the extract (150, 300, and 600 mg/kg body weight) were prepared for oral administration. The required quantity of C. asiatica powder for each dose was mixed with 6 mL distilled water according to previously described methods [19, 20]. Distilled water was first boiled and then allowed to cool to approximately 85 °C before extraction. The powdered plant material was gradually added to the warm water under continuous stirring to maintain proper homogenization. After complete mixing, the suspension was transferred into a flask covered with aluminum foil to minimize light exposure and preserve phytochemical stability. The preparation was stored in a dark place at room temperature for 24 h to facilitate aqueous extraction [22, 23, 24]. Following extraction, the mixture was filtered through filter paper, and the obtained filtrate was used immediately for oral administration to experimental animals. Freshly prepared extract was used to ensure consistency and stability of the phytochemical constituents.

Phytochemical Characterization of C.asiatica Extract Preliminary qualitative phytochemical screening of the aqueous C.asiatica extract was performed using standard phytochemical methods to identify major bioactive constituents. The extract was screened for flavonoids, phenolic compounds, tannins, saponins, alkaloids, and triterpenoids. The analysis confirmed the presence of flavonoids, phenolics, saponins, and triterpenoids in the aqueous extract, indicating the presence of bioactive compounds that may contribute to the observed antioxidant and gastroprotective activities.

### Preparation of Omeprazole Solution

Omeprazole was used as the reference anti-ulcer drug because of its established gastroprotective activity in gastric ulcer disease [25]. One omeprazole capsule containing 20 mg active ingredient was dissolved in 7 mL distilled water immediately before administration. The prepared solution was administered orally to the omeprazole-treated group under the same experimental conditions applied to the other groups.

### Animals and Ethical Approval

Healthy adult male Sprague-Dawley rats weighing 190-220 g were obtained from the Experimental Animal House, College of Applied Science, Cihan University-Erbil, Iraq. The experimental protocol was approved by the Ethics Committee of Cihan University-Erbil for Animal Experimentation (Approval No.: CUE-REC/2025/10). All experimental procedures were conducted according to the National Research Council Guide for the Care and Use of Laboratory Animals and NIH guidelines. Initially, 32 rats were enrolled in the study. During acclimatization and experimental handling, eight animals were excluded because of stress-related complications and failure to satisfy inclusion criteria before treatment allocation. Therefore, 24 rats completed the experimental protocol and were included in the final analysis. Animals were randomly allocated into five experimental groups using a simple randomization technique, with six rats in each group. During the experimental period, rats were housed individually in cages with wide-mesh wire floors to minimize coprophagia. Standard pellet chow and tap water were supplied ad libitum except during fasting before ulcer induction. Animals were randomly allocated into experimental groups using a simple randomization method. Histopathological evaluation was performed in a blinded manner to minimize observational bias during tissue assessment.

### Induction of Ethanol-Induced Gastric Ulcers

Rats were fasted for 48 h before ulcer induction, while water was permitted until 2 h before the procedure. Gastric ulceration was induced by oral administration of absolute ethanol (5 mL/kg) using a modified experimental method described previously [26]. Oral gavage was selected because it provides direct and reproducible exposure of the gastric mucosa to ethanol and is widely used in experimental models of acute gastric ulceration. The ulcer control group received absolute ethanol alone and served as the untreated ulcerated group. The reference treatment group received omeprazole (20 mg/kg dissolved in distilled water), whereas the experimental groups received aqueous C.asiatica extract at doses of 150, 300, and 600 mg/kg body weight orally. All pretreatments were administered once by oral gavage. One hour after pretreatment, all animals received absolute ethanol (5 mL/kg orally) to induce gastric mucosal injury. Sixty minutes after ethanol administration, rats were deeply anesthetized using ketamine/xylazine overdose. Blood samples were collected aseptically via cardiac puncture approximately 10 minutes after induction of anesthesia. Animals were then euthanized by cervical dislocation, and the stomachs were immediately removed for macroscopic, histopathological, and molecular investigations [27]. Macroscopic Assessment of Gastric Lesions After sacrifice, stomachs were excised and opened along the greater curvature. Gastric contents and blood clots were gently removed using normal saline without causing additional mucosal injury. The gastric mucosa was examined macroscopically for visible hemorrhagic lesions, which appeared as elongated bands parallel to the longitudinal axis of the stomach. Ulcer dimensions including length, width, and lesion extent were measured using a planimeter according to previously described methods [29, 30]. For more accurate estimation of ulcer area, a transparent 2 × 2 mm grid was placed over the gastric mucosa, and the number of squares covering the lesion area was counted. The ulcer area (UA) for each stomach was calculated by multiplying the total number of counted squares by 4 and then by 1.8 to obtain the final value in mm². Percentage ulcer inhibition (I%) was calculated using the following formula: I% = [(UA control - UA treated) / UA control] × 100

### Histopathological Examination of Gastric Tissue

Immediately after sacrifice, gastric tissue samples were carefully rinsed with normal saline to remove residual blood and gastric contents. Representative sections of gastric tissue were fixed in 10% buffered formalin for preservation of tissue architecture. Following fixation, tissues were processed routinely, embedded in paraffin wax, sectioned at 5 µm thickness, and stained with hematoxylin and eosin (H&E). Histopathological examination was performed under light microscopy by a blinded histopathologist. Microscopic evaluation included assessment of epithelial disruption, mucosal necrosis, hemorrhage, edema, and inflammatory-cell infiltration.

### Estimation of Gastric Mucus Weight

After opening the stomach along the greater curvature, gastric mucus adherent to the mucosal surface was carefully collected into pre-weighed containers. Special care was taken to avoid unnecessary loss of mucus during sample collection to ensure consistency among samples. Gastric mucus weight was determined by subtracting the weight of the empty container from the final sample weight. The measured value represented gastric mucus secretion and was considered an indirect indicator of gastric mucosal defence activity.

### Measurement of Gastric pH

Gastric pH was measured to evaluate intragastric acidity and secretory activity of the gastric mucosa. After opening the stomach, gastric contents including the mucus layer were collected, and pH was measured immediately using a calibrated digital pH meter. Immediate measurement minimized variations related to sample handling and environmental exposure. Gastric pH was used as an indicator of mucosal defensive status because the mucus layer contributes to bicarbonate retention and maintenance of epithelial integrity.

Quantitative Real-Time PCR Analysis of Antioxidant-Related Genes (Nrf2 and HO-1) Total RNA was extracted from gastric tissue samples obtained from the control group, ethanol-treated group, omeprazole-treated group (20 mg/kg), and Centella asiatica-treated groups (150, 300, and 600 mg/kg) using TRIzol RNA Extraction Reagent (Thermo Fisher Scientific, Waltham, MA, USA). RNA concentration and purity were determined spectrophotometrically using a NanoDrop 2000 spectrophotometer (Thermo Fisher Scientific, USA), and samples with A260/A280 ratios of approximately 2.0 were considered suitable for downstream molecular analysis. Complementary DNA (cDNA) synthesis was performed using 1 µg of total RNA with the High-Capacity cDNA Reverse Transcription Kit (Applied Biosystems, Foster City, CA, USA) according to the manufacturer’s instructions. Quantitative real-time PCR analysis was carried out using SYBR Green Master Mix (Applied Biosystems, Foster City, CA, USA) on a StepOnePlus Real-Time PCR System (Applied Biosystems, USA). Gene-specific primers were used for Nrf2 and HO-1, while Gapdh and Actb were used as housekeeping reference genes for normalization. Normalization of gene-expression levels was performed using the geometric mean of the Ct values of Gapdh and Actb. Amplification specificity was confirmed by melt-curve analysis, which demonstrated a single amplification product for each target gene, and amplification efficiency for all primer sets ranged between 95% and 105%. Each sample was analyzed in technical duplicate. Relative gene-expression levels were calculated using the 2^-ΔΔCt method, with the ethanol-treated group serving as the calibrator. Results were expressed as mean ± standard deviation (SD) for six animals per group [28]. Lower ΔCt values indicate relatively higher target gene expression levels because fewer amplification cycles are required to reach the detection threshold. Relative gene expression was subsequently calculated using the 2^-ΔΔCt method.

### Statistical Analysis

Statistical analysis was performed using GraphPad Prism version 10.4.1 (GraphPad Software, San Diego, CA, USA). Data were expressed as mean ± standard deviation (SD). Normality of data distribution was assessed using the Shapiro-Wilk test, while homogeneity of variance was evaluated using Levene’s test. Comparisons among experimental groups were performed using one-way analysis of variance (ANOVA) followed by Tukey’s multiple-comparison post hoc test. Pairwise comparisons between the omeprazole-treated group and the Centella asiatica-treated groups were also analyzed. Differences were considered statistically significant at P<0.05.

## Results

**Table:** Table1. Gastric pH, Mucus Weight, Ulcer Area, and Ulcer Inhibition Percentage Across Experimental Groups

Group	Gastric pH	Gastric Mucus Weight (g)	Ulcer Area (mm²)	Ulcer Inhibition (%)
Ulcer control	2.62	0.77	822	—
Omeprazole (20 mg/kg)	4.97	2.47	125	84.79
C.asiatica (150 mg/kg)	4.45	1.97	76	90.75
C.asiatica (300 mg/kg)	5.42	2.24	61	92.57
C.asiatica (600 mg/kg)	6.42	2.34	9.2	98.88

**Figure-1:**
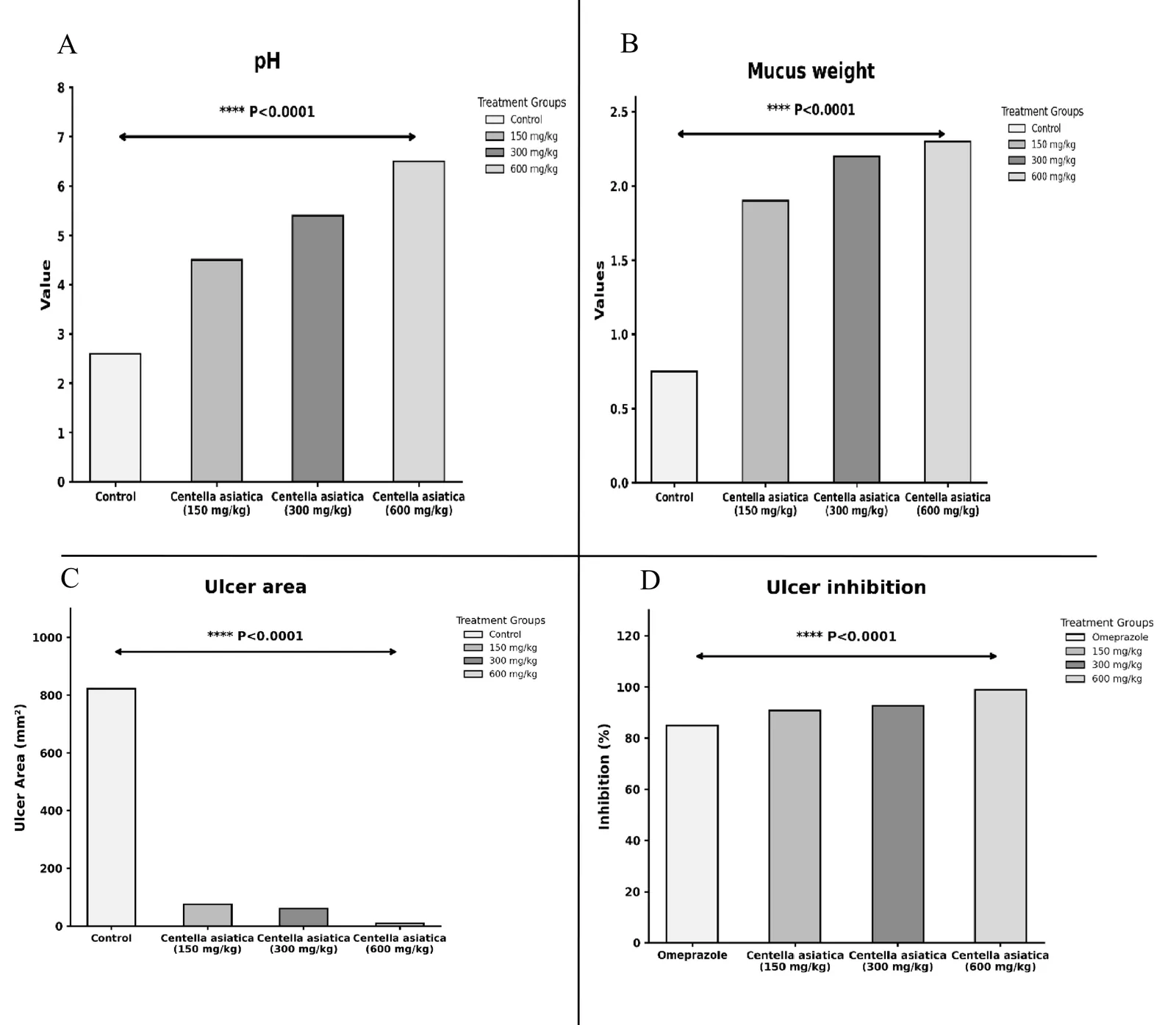
Figure 1. Effects of aqueous Centella asiatica extract on gastric parameters in ethanol-induced gastric ulcer rats. (A) Effect of C. asiatica treatment at different doses on gastric pH levels. (B) Effect of aqueous C. asiatica extract on gastric mucus weight. (C) Effect of aqueous C. asiatica extract on ulcer area. (D) Effect of aqueous C. asiatica extract on ulcer inhibition percentage.

**Figure-2:**
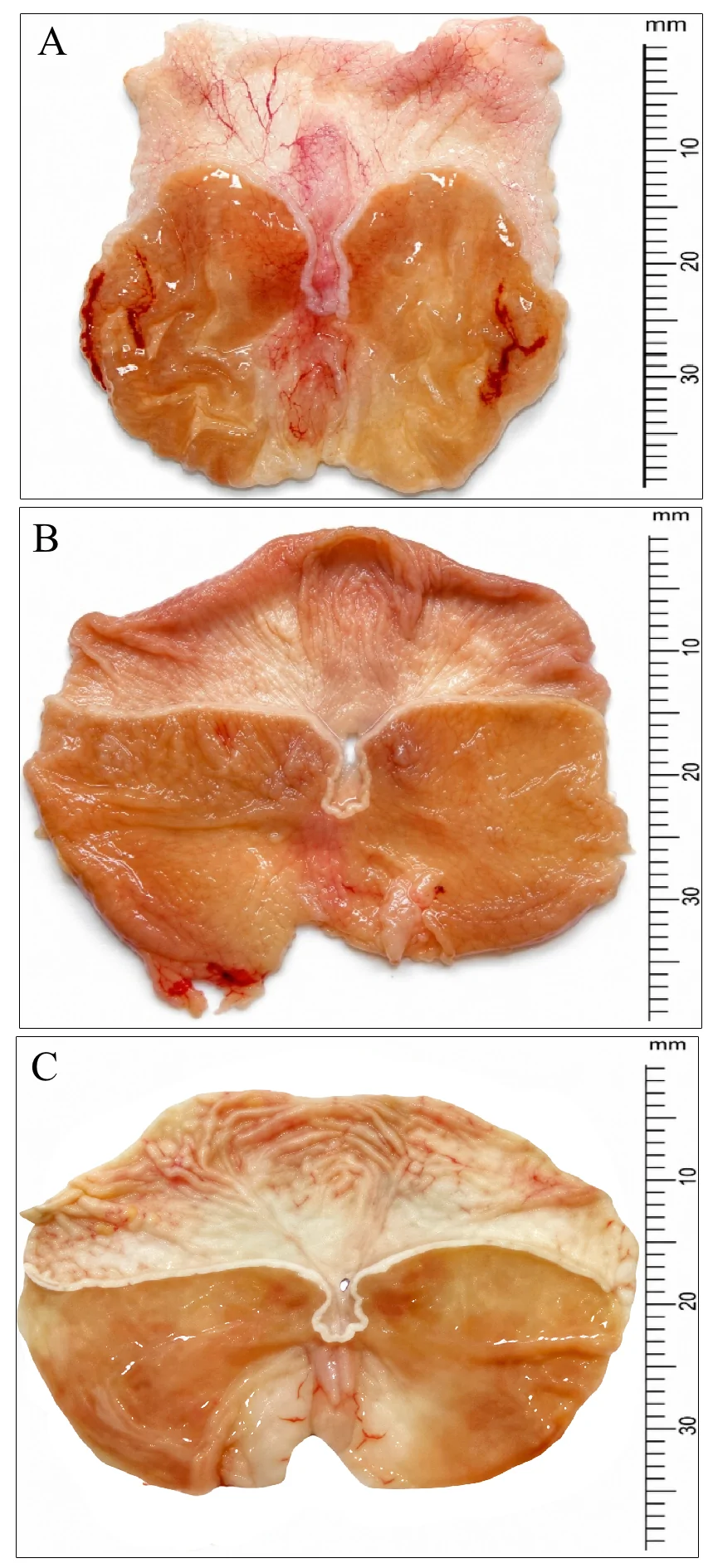
Figure 2. Representative gross morphology of gastric mucosa in different experimental groups. (A) Ulcer control group showing extensive hemorrhagic lesions and severe gastric mucosal injury induced by ethanol administration. (B) Omeprazole-pretreated group (20 mg/kg) showing reduced hemorrhagic lesions and preservation of gastric mucosal integrity. (C) Aqueous C.asiatica extract-pretreated group (600 mg/kg) showing marked protection against ethanol-induced gastric injury with minimal visible lesions.

**Figure-3:**
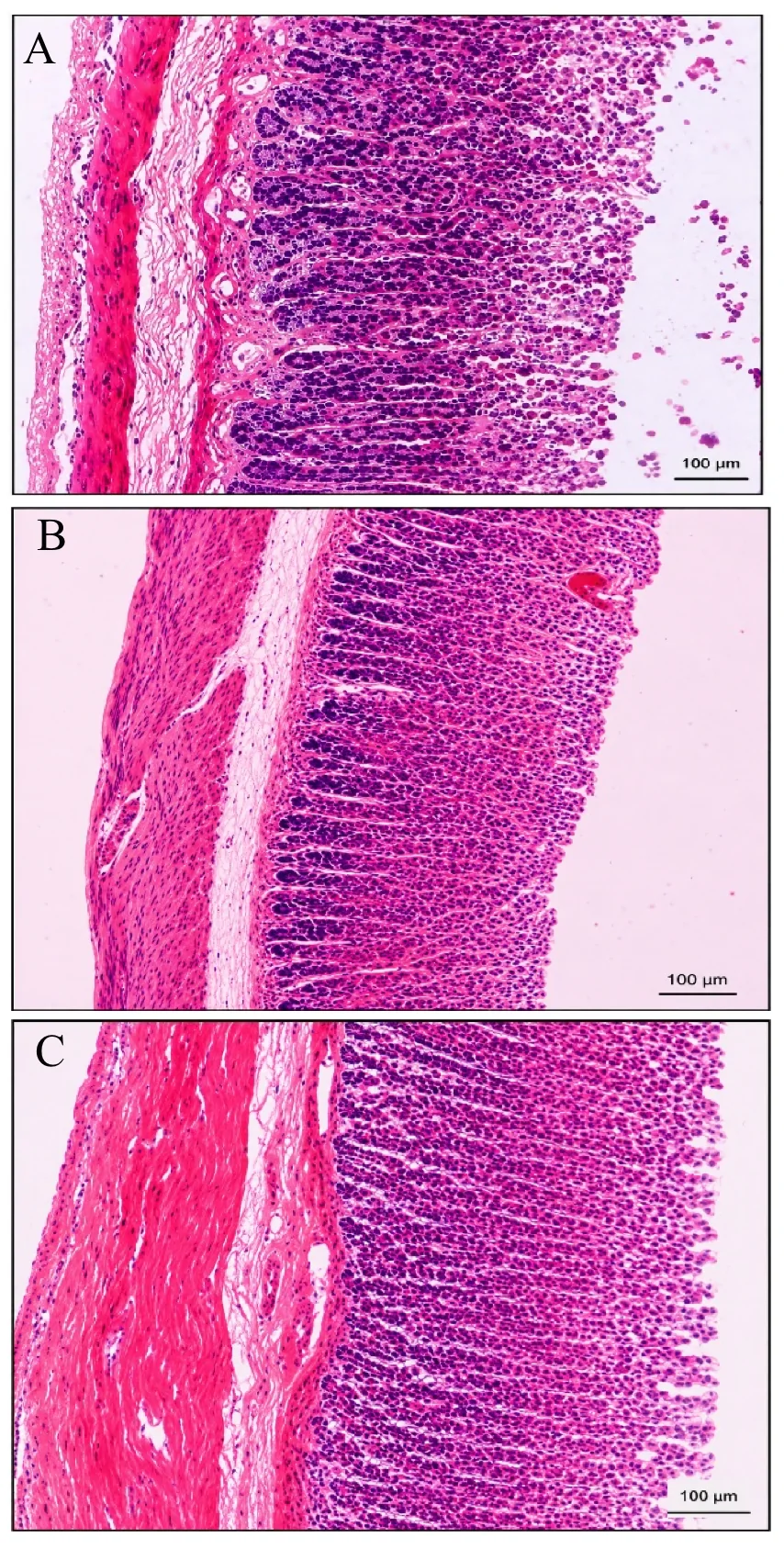
Figure 3. Histopathological section of gastric tissue from the ulcer in different experimental groups stained with hematoxylin and eosin (H&E), showing severe epithelial disruption, submucosal edema, hemorrhage, and inflammatory-cell infiltration (100×). (A) control group, (B) Omeprazole-pretreated group (20 mg/kg) , and (C) C.asiatica extract-treated group (600 mg/kg)

**Figure-4:**
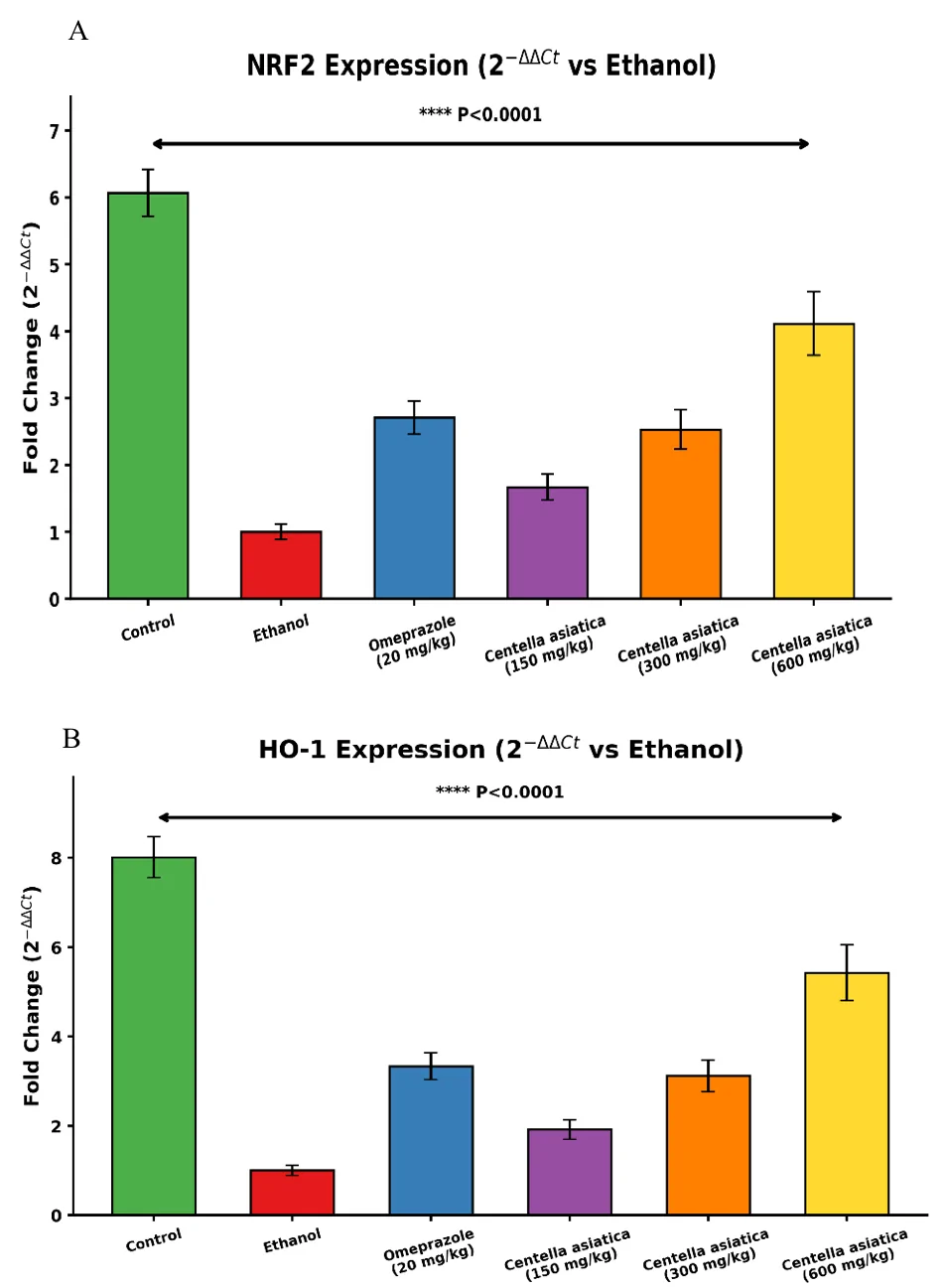
Figure 4. Relative expression of antioxidant-related genes in gastric tissue following pretreatment with aqueous Centella asiatica extract and omeprazole in ethanol-induced gastric ulcer rats. (A) Relative expression of Nrf2. (B) Relative expression of HO-1. Data are expressed as fold change (2^−ΔΔCt) relative to the ethanol-treated group and presented as mean ± SD (n=6). ****P<0.0001 versus the ethanol-treated group

### Acute Toxicity Assessment

Acute toxicity evaluation was performed to assess the safety of aqueous C.asiatica extract at doses of 150, 300, and 600 mg/kg in experimental rats. During the 14-day observation period, no mortality or visible signs of toxicity were observed in any treatment group. Animals maintained normal behavior and showed no significant abnormalities in body-weight progression throughout the study period. Gross examination did not reveal any remarkable pathological changes in the examined organs. Hematological and serum biochemical parameters, including triglycerides, creatinine, urea, hemoglobin (HGB), aspartate aminotransferase (AST), alanine aminotransferase (ALT), and alkaline phosphatase (ALP), were comparable between extract-treated and normal control animals, with no statistically significant differences observed (P>0.05). These findings indicate that aqueous C.asiatica extract was well tolerated at the investigated doses and did not produce significant acute toxic effects under the present experimental conditions.

### Gross Evaluation of Gastric Lesions

The gastroprotective effects of aqueous C.asiatica extract against ethanol-induced gastric mucosal injury are summarized in Table1. Pretreatment with aqueous C.asiatica extract significantly reduced the severity of ethanol-induced gastric injury in a dose-dependent manner compared with the ulcer control group (P<0.05). Improvements were observed in gastric pH, gastric mucus weight, ulcer area, and ulcer inhibition percentage (Figure-1). The ulcer control group demonstrated severe gastric mucosal injury characterized by extensive hemorrhagic lesions and the largest ulcer area (Figure-2 A). In contrast, pretreatment with omeprazole or aqueous C. asiatica extract reduced the severity of visible gastric lesions and improved gastric mucosal appearance (Figure-2 B and C). The gastroprotective effect became progressively more pronounced with increasing extract dose. The group treated with 600 mg/kg aqueous C.asiatica extract demonstrated the greatest protective effect, with ulcer area reduced to 9.2 mm² and ulcer inhibition reaching 98.88%, which exceeded the protection observed in the omeprazole-treated group. Macroscopic examination of excised stomach tissues supported these findings (Figure-2). The ulcer control stomachs exhibited extensive mucosal injury, whereas extract-treated groups demonstrated progressive preservation of gastric mucosal integrity with reduced lesion severity. No obvious gross mucosal lesions were observed in the group treated with 600 mg/kg aqueous C.asiatica extract. Overall, aqueous C.asiatica extract significantly attenuated ethanol-induced gastric mucosal injury and demonstrated marked dose-dependent gastroprotective activity. Histopathological Evaluation of Gastric Lesions Histopathological examination of gastric tissues from the ulcer control group demonstrated severe mucosal injury characterized by extensive epithelial disruption, marked submucosal edema, hemorrhagic changes, inflammatory-cell infiltration, and considerable destruction of gastric mucosal architecture following ethanol administration. In contrast, pretreatment with aqueous C.asiatica extract produced marked histopathological improvement compared with the ulcer control group, as evidenced by better preservation of mucosal architecture, reduced lesion severity, minimal submucosal edema, and decreased inflammatory-cell infiltration (Figure-3 A). The omeprazole-pretreated group also demonstrated reduced histopathological damage compared with the ulcer control group (Figure-3 B). The protective effect of aqueous C.asiatica extract became progressively more pronounced with increasing dose, and the 600 mg/kg treatment group exhibited the greatest preservation of normal gastric histological structure with minimal evidence of mucosal injury (Figure-3 C). These histopathological findings were consistent with the gross macroscopic observations and further supported the gastroprotective activity of aqueous C.asiatica extract against ethanol-induced gastric mucosal injury. Gene Expression of Nrf2 and HO-1 Expression levels of the antioxidant-response genes Nrf2 and HO-1 were significantly reduced following ethanol administration compared with the normal control group (Figure-4). Pretreatment with omeprazole and aqueous C.asiatica extract partially restored the expression of both genes in a dose-dependent manner. For Nrf2, relative fold-change expression values (2^-ΔΔCt using the ethanol-treated group as calibrator) were 6.071 ± 0.349 in the normal control group, 1.005 ± 0.115 in the ethanol-treated group, and 2.710 ± 0.244 in the omeprazole-pretreated group, whereas aqueous C.asiatica-treated groups demonstrated values of 1.671 ± 0.192, 2.533 ± 0.291, and 4.114 ± 0.473 at doses of 150, 300, and 600 mg/kg, respectively. A similar expression pattern was observed for HO-1. Relative fold-change values were 8.011 ± 0.461 in the normal control group, 1.005 ± 0.115 in the ethanol-treated group, and 3.336 ± 0.300 in the omeprazole-pretreated group, while aqueous C.asiatica-treated groups demonstrated HO-1 expression values of 1.919 ± 0.221, 3.118 ± 0.358, and 5.429 ± 0.624 at doses of 150, 300, and 600 mg/kg, respectively. Overall, pretreatment with aqueous C.asiatica extract increased Nrf2 and HO-1 expression in a dose-dependent manner, with the highest expression observed in the 600 mg/kg treatment group.

## Discussion

Gastric ulceration is a multifactorial inflammatory condition that results from an imbalance between aggressive factors and the protective mechanisms of the gastric mucosa [29, 30]. Although gastric acid and pepsin contribute to mucosal injury, disruption of the mucus-bicarbonate barrier, impaired mucosal blood flow, oxidative stress, and inflammatory responses play important roles in ulcer development. In the present study, ethanol administration produced severe gastric mucosal injury characterized by extensive hemorrhagic lesions, epithelial disruption, submucosal edema, inflammatory-cell infiltration, reduced gastric pH, decreased mucus weight, and increased ulcer area. These findings are consistent with previous reports demonstrating the damaging effects of ethanol on gastric mucosal integrity through direct epithelial injury and inflammatory responses [30, 31].

Pretreatment with aqueous C.asiatica extract significantly attenuated ethanol-induced gastric injury in a dose-dependent manner [32]. The extract reduced ulcer area, increased ulcer inhibition percentage, improved gastric pH, and enhanced gastric mucus production compared with the ulcer control group [33]. Among the tested doses, 600 mg/kg demonstrated the greatest gastroprotective effect, with marked preservation of gastric mucosal integrity [33, 34]. Histopathological findings further supported these observations, as extract-treated groups showed reduced epithelial disruption, minimal submucosal edema, and decreased inflammatory-cell infiltration compared with untreated ulcerated animals [31]. The protective effect observed with the highest dose of aqueous C. asiatica extract was comparable to that observed in the omeprazole-treated group [29].

Enhancement of gastric mucus production may represent an important protective mechanism of the extract. Gastric mucus acts as a physical and chemical barrier that protects the epithelium against luminal irritants and maintains bicarbonate concentration at the mucosal surface [30]. In addition, increased gastric pH observed in extract-treated groups may have contributed to reduction of mucosal irritation and preservation of epithelial integrity [30].

The present study also demonstrated that ethanol administration markedly reduced the expression of the antioxidant-response genes Nrf2 and HO-1, whereas pretreatment with aqueous C.asiatica extract restored their expression in a dose-dependent manner (Figure . The highest gene-expression levels were observed in the 600 mg/kg treatment group. Because Nrf2 and HO-1 are important regulators of cellular antioxidant defense and cytoprotective responses, restoration of their expression may contribute to the gastroprotective activity observed in the treated groups [31].

Although minor variations were observed between the histopathological, biochemical, and molecular findings, the overall results demonstrated a consistent protective trend associated with aqueous C.asiatica treatment. Histological improvement was more pronounced at higher extract doses, whereas some molecular and functional parameters showed gradual dose-dependent responses. Such variations may reflect differences in the sensitivity and timing of tissue injury, inflammatory responses, and antioxidant gene activation following ethanol exposure. Nevertheless, the collective findings from gross morphology, gastric mucus production, gastric pH, histopathological evaluation, and Nrf2/HO-1 gene expression support the gastroprotective activity of aqueous C.asiatica extract in this experimental model [32, 33, 34].

The gastroprotective effects of C. asiatica may be related to its phytochemical constituents, including triterpenoids, flavonoids, phenolic compounds, and saponins, which have been reported to possess antioxidant and anti-inflammatory activities [32, 33, 34, 35]. Preliminary phytochemical screening in the present study confirmed the presence of flavonoids, phenolics, saponins, and triterpenoids in the aqueous extract. Although individual bioactive compounds were not quantitatively analyzed, the observed improvements in gastric morphology, mucus secretion, histopathological appearance, and antioxidant-related gene expression collectively support the protective potential of the aqueous extract [30, 31, 32, 33].

Acute toxicity assessment demonstrated that aqueous C.asiatica extract was well tolerated at the investigated doses, with no mortality or significant toxic effects observed during the experimental period [33]. Overall, the findings of this study demonstrate that aqueous C.asiatica extract exerts significant dose-dependent gastroprotective effects against ethanol-induced gastric mucosal injury, with the greatest protective activity observed at 600 mg/kg. Further studies are recommended to isolate and quantify the active phytochemical constituents and to clarify the molecular mechanisms underlying its gastroprotective activity [31, 32, 34].

## Conclusion

The present study demonstrated that aqueous C.asiatica extract exerted significant dose-dependent gastroprotective effects against ethanol-induced gastric mucosal injury in rats. Pretreatment with the extract reduced ulcer area, increased gastric mucus production and gastric pH, improved histopathological appearance, and enhanced the expression of the antioxidant-response genes Nrf2 and HO-1. The greatest protective effect was observed at the 600 mg/kg dose. These findings suggest that aqueous C. asiatica extract may contribute to gastric mucosal protection through enhancement of mucosal defense and antioxidant-response mechanisms. Further studies are required to identify the active phytochemical constituents and clarify the molecular pathways involved in its gastroprotective activity.

## Conflict of Interest

The authors declare that there are no conflicts of interest regarding the publication of this study.

## AI Disclosure Statement

During the preparation of this work, the authors used Grammarly and Zotero for language editing, grammar correction, reference management, and formatting support. After using these tools, the authors reviewed and edited the manuscript as needed and take full responsibility for the content of the publication.
